# Seprafilm® adhesion barrier: (1) a review of preclinical, animal, and human investigational studies

**DOI:** 10.1007/s10397-012-0741-9

**Published:** 2012-04-20

**Authors:** Michael P. Diamond, Ellen L. Burns, Beverly Accomando, Sadiqa Mian, Lena Holmdahl

**Affiliations:** 1 Division of Reproductive Endocrinology and Infertility, Department of Obstetrics and Gynecology, Wayne State University, 60 West Hancock, Detroit, MI 48201 USA; 2 Genzyme Corporation, Cambridge, MA USA

**Keywords:** Postoperative adhesions, Seprafilm, Anti-adhesion adjuvant, Adhesiolysis

## Abstract

The aim of this study was to provide a single site resource for investigators, clinicians, and others seeking preclinical, animal, and human investigational studies concerning the postsurgical, anti-adhesion barrier Seprafilm™ (Genzyme Corporation, Cambridge, MA). All published preclinical, animal, human extra-abdominal research as of July 2011 have been summarized and included in this document. Searches of Medline and EMBASE Drugs and Pharmaceuticals databases were conducted for original preclinical, animal, and human extra-abdominal studies involving Seprafilm. Preclinical, animal, and extra-abdominal human investigational studies are the study selection for this manuscript. Intraabdominal use is discussed in the accompanying manuscript. Data extraction includes systematic manuscript review. Summary of preclinical, animal, and extra-abdominal human investigational use of Seprafilm by surgical discipline were gathered for data synthesis. The clinical use of Seprafilm, which was approved by the FDA for intra-abdominal procedures, is supported by preclinical and animal studies relating to general surgical and obstetrical/gynecological applications. Findings from preclinical, animal, and human investigational studies at other sites throughout the body raises the potential for additional human clinical trials to assess efficacy and safety following surgical procedures at non-abdominal locations.

## Introduction

Postoperative adhesions lead to significant patient morbidity such as small bowel obstruction, infertility, chronic pain, and difficult, complicated subsequent surgeries. Various strategies have been put forth to prevent adhesion development including meticulous surgical techniques, pharmacological agents, and new equipment and instrumentation. Yet, despite these interventions, the overwhelming majority of patients continue to develop adhesions after surgical procedures.

## Pathogenesis of adhesions

Considerations for adhesion prevention should be based upon an understanding of peritoneal repair, and what goes awry when adhesions develop, as briefly summarized below [1–3]. Postoperative adhesion development is teleologically considered to be an effort by the body to restore the supply of oxygen and nutrients to tissue which has been injured during the surgical procedure. As a sequelae to tissue injury, an inflammatory-like response occurs in response to surgery-induced tissue hypoxia, with resultant generation of reactive oxygen and reactive nitrogen species. This inflammatory response invokes release of histamine, cytokines, and growth factors; infiltration of polymorphonuclear leukocytes; and the migration of fibroblasts from underlying tissues. Collections of blood and serosanguinous tissue exudates occur on the surface of injured tissue forming a fibrinous mass. In the presence of diminished or absent tissue plasminogen activator activity, the fibrinous mass persists long enough for infiltration by the migrating fibroblasts from the underlying tissues, with subsequent deposition of extracellular matrix including collagen and fibronectin. Persistence of relative hypoxia induces vascular endothelial growth production with subsequent angiogenesis. This process persists during and beyond the 3 to 5 days required for remesotheliazation of injured tissue surfaces.

If this concept of adhesion development is correct, then the critical time to prevent or diminish adhesion development is in the initial 3 to 5 days after the surgical procedure, prior to completion of remesotheliazation. Additionally, adjuvants and approaches to reduce adhesions would either interrupt the molecular biologic process leading to adhesion development and/or physically separate opposing tissue surfaces at risk for adhesion development until after remesotheliazation is complete. It is this latter approach which has been utilized by all of the FDA-approved devices for the reduction of postoperative intra-abdominal and pelvic adhesions, including Seprafilm. This manuscript summarizes the preclinical, animal, and human investigational studies involving Seprafilm. A companion manuscript summarizes reports of intra-abdominal use of Seprafilm for general surgical and gynecological operative procedures.

## Seprafilm adhesion barrier

### Physical properties and clearance

Seprafilm is a bioresorbable membrane composed of sodium hyaluronate (HA) and carboxymethylcellulose (CMC) which have been chemically modified to delay the rate of degradation and clearance after placement in the body. Seprafilm is applied to the surface of tissues to be protected and hydrates to form a lubricious gel coating within 24 to 48 h of placement. The hydrophilic protective gel acts as physical barrier to separate adjacent serosal tissues during the critical stages of wound repair and has no pharmacological effect. During the transition from a solid to gel, the volume of the barrier increases, but its swelling pressure decreases from 6.4 atm to 0. In addition, Seprafilm’s tensile strength decreases by 90 % within 30 min. The membrane’s swelling does not affect peritoneal tissue or organ function. The barrier is resorbed from the site of application within 7 days and therefore does not require a second operation for removal. Radiolabeled studies of Seprafilm showed that it is totally cleared from the body within 28 days following implantation. Seprafilm is excreted primarily through the kidneys. Seprafilm’s rapid degradation reduces the risk of a foreign body tissue response [4].

### Preclinical safety and efficacy studies

The safe and effective use of Seprafilm in the abdominal cavity of animals has been reported in a number of publications using multiple species and models of abdominal surgery [4–34]. In these models, the use of Seprafilm was reported to be efficacious in reducing adhesions, including in the presence of blood and irrigation solution [4], in combination with melatonin [33] and under ischemic conditions [4]. When used in multiple layers [4], Seprafilm did not adversely affect wound healing [4, 6, 8, 35], did not affect anastomotic healing with [36] or without concomitant ischemia [35, 37], and did not potentiate intra-abdominal sepsis [11, 38]. In general, Seprafilm use has been reported not to have adverse effects [5, 7, 9, 10, 12, 13, 39–41]. Concomitant radiotherapy did not alter the efficacy [42]; however, the efficacy of Seprafilm was reduced in the presence of peritonitis [43].

In addition, the use of Seprafilm in animal models has been reported from a number of anatomical sites outside of the abdominal cavity, including following nerve repair [44] and cancer treatment [45–48]. In this series of articles, the application of Seprafilm did not increase cancer cell growth, wound site implantation, or distant metastasis and did not decrease host survival. Additionally, Seprafilm has been reported to be safe and beneficial when used in conjunction with cardiac surgery [49–51], tendon surgery [52], joint surgery [53], spinal surgery [54–56], strabismus surgery [57], and tympanic membrane surgery [58].

Multiple preclinical safety studies in various animal models have been conducted to assess the immunogenicity, biocompatibility, and tissue response to Seprafilm [4]. Dermal and systemic immunogenicity (using sodium hyaluronate–carboxymethylcellulose (HA-CMC) extracts) were studied with two series of intradermal injections in 15 adult albino guinea pigs, and by six intraperitoneal injections in ten adult guinea pigs over a period of 2 weeks followed by an intravenous challenge injection, respectively [4]. Dermal injection sites were observed for erythema and edema over a 96-h period; no indications of dermal sensitization were observed. In addition, no systemic response suggestive of antigenecity or anaphylaxis was observed after multiple intraperitoneal injections followed by an intravenous challenge injection.

Biocompatibility was assessed through studies of in vitro hemolysis, in vitro complement activation, in vitro cytotoxicity, and muscle implant tests [4]. In all cases, Seprafilm did not elicit reactions suggestive of tissue incompatibility. Tissue response to Seprafilm was examined in a rat model by abrading the rat cecum, followed by either the application of Seprafilm (treatment group) or no further intervention (untreated group). Animals were observed for 28 days; inflammatory cells including macrophages were noted at day 2 in both groups. At day 4, a large number of macrophages (no giant cells) were observed in the treatment group suggestive of HA-CMC degradation, while the untreated group displayed fewer macrophages and occasional polymorphonuclear cells, suggestive of the normal wound healing process. At day 28, the rat cecum appeared to be healed in both groups, but the untreated group had denser granulation tissue at the wound site. Additionally, the Ames mutagenicity test, the rabbit pyrogen test, the intracutaneous toxicity test, and the acute intraperitoneal and systemic toxicity tests, respectively demonstrated that Seprafilm is biocompatible, non-mutagenic, non-pyrogenic, non-irritating, and nontoxic [4].

Preclinical evaluation of the efficacy of Seprafilm in a rat cecal abrasion or side wall injury model demonstrated that Seprafilm significantly reduced the incidence of cecal adhesions (*P* < 0.001) and the number of animals with severe adhesions (*P* < 0.001) when compared with non-treated controls [4]. This efficacy was maintained in the presence of blood, excess irrigation fluids, and layering of Seprafilm, as well as under ischemic conditions. Subsequently, in cell culture studies of human peritoneal and adhesion fibroblasts, Seprafilm was not observed to alter expression of inflammatory markers such as type I collagen and transforming growth factor beta [59].

Efficacy of Seprafilm has also been demonstrated in an adhesion reformation model [33]. In this study, adhesion reformation was induced between the uterine horns by creation of ten standardized lesions on the uterine horns using bipolar electrocoagulation, with separation from tissue lying above and below with Seprafilm. Adhesiolysis was performed at the time of a second-look procedure with randomization to one of four groups: control, Seprafilm alone, melatonin (a free radical scavenger) alone (1 ml of a 2-mg/ml solution), or a combination of Seprafilm plus melatonin. At the time of a third-look procedure, adhesion reformation was significantly reduced by either Seprafilm or melatonin alone compared to control animals, with a further significantly greater reduction in adhesion reformation achieved with the combination of Seprafilm plus melatonin [33].

### Use of Seprafilm with specific conditions

#### Infection

The effect of Seprafilm on abscess development was studied in three independent animal studies in which Seprafilm was placed in the presence of existing peritonitis. In two of those studies, Seprafilm had no effect on abscess development [60] while in a third study Seprafilm was associated with increased abscess development when placed in the presence of ongoing peritonitis [43]. Although these results are inconsistent, the Seprafilm label directs users not to use the product in the presence of frank infection. Additional work in experimental models have shown that Seprafilm had no effect on sepsis or abscess development when Seprafilm placement was concomitant with bacterial contamination [38] when placed in a surgical model of bowel injury [11] and that it did not exaggerate intra-abdominal septic conditions or induce a systematic inflammatory response [61].

To further elucidate mechanistic aspects of the possible impact of Seprafilm on infectious processes, Otake et al. examined the effect of Seprafilm in polymorphonuclear (PMN) obtained from 14 subjects [62]. Seprafilm was evaluated in two forms, as a film and after it was well dissolved and compared to corresponding controls. Seprafilm, in either form, had no effect on the rate of phagocytosis of *Escherichia coli* or *Staphylococcus aureus*, the rate of PMN-induced apoptosis of *E. coli* and *S. aureus*, and the effect of PMN-induced necrosis of *E. coli* and *S. aureus*. Examining cytokine production when co-cultured with *E. coli* or *S. aureus*, Seprafilm also had no effect on production of the cytokines IL-1α, IL-6, or IL-8 or on the production of IL-1 RA or PMN elastase. There was also no effect on PMN cytokine production when stimulated with TNF-α or LPS.

#### Malignancy

In vitro studies did not find any increase in the growth of colon cancer cells when cultured in the presence of Seprafilm [45]. Preclinical studies in mouse or hamster models did not observe any increase in cancer cell growth, wound site implantation, distant metastasis, or host survival with the use of Seprafilm in the presence of cancer cells, when compared with the control [46–48].

### Animal investigational studies of use of Seprafilm

#### Cardiac surgery

Preclinical studies in dogs, pigs, sheep, and rabbits evaluating the use of Seprafilm in cardiac surgery observed a significant decrease in the severity of pericardial adhesions in most [49–51, 63], but not all studies [64].

#### Thoracic surgery

A preclinical study in sheep was done to assess the effect of Seprafilm on pleural adhesion formation after experimental thoracotomy and parietal pleurectomy [65]. A significant decrease in the incidence (*P* < 0.001) and severity (*P* < 0.001) of the pleural adhesions was noted in the Seprafilm group when compared with control. There was no increase in inflammation in the areas of Seprafilm application. A preclinical study in rats was unable to identify a benefit or detriment when the esophagus was wrapped with Seprafilm or Interceed, as assessed by adhesion score 3 weeks later or by hyroxyproline levels [66].

#### Vascular surgery

To investigate whether external support would reduce intimal hyperplasia at the site of venous graft anastamosis, rabbits undergoing bypass grafting of the right jugular vein to the right common carotid artery was performed in the presence or absence of wrapping the anastamosis with Seprafilm [67]. Evaluation at 1 month demonstrated that use of Seprafilm reduced both neointimal and medial thickening.

#### Orthopedic surgery

In a preclinical study, Seprafilm was used to prevent adhesions after tenolysis at the flexor digitorum tendon in chickens [52]. Six weeks after the application of Seprafilm, tendons that received Seprafilm were seen to have a significantly improved gliding excursion profile (*P* < 0.05) and decreased incidence of adhesions (*P* < 0.05), when compared with controls. Consistent results were observed in a chicken gastrocnemius tendon model, in which Seprafilm wrapping resulted in improved tendon sheath complex sliding, less histological evidence of tissue inflammation, and greater tendon tensile strength, as compared to untreated controls [68]. Similarly, in a rabbit partial-thickness flexor tendon injury–suture repair model, comparing Seprafilm and other HA-based products versus controls, there was a reduction in both the gross adhesion score and the histologic adhesion score and an improvement in histologic assessment of tender healing [69]. In a biomechanical test, the force required to remove the tendon from its sheath was less for Seprafilm than injured, untreated control animals.

The safety of Seprafilm use in femorotibial joints was studied in adult female New Zealand White rabbits [53]. Intraarticular administration of Seprafilm in femorotibial joints of rabbits was associated with transient uptake by synovial phagocytes, but no local toxicity was apparent for up to 6 months.

Three studies have evaluated Seprafilm after laminectomy. The first preclinical study compared the effect of Seprafilm and GORE-TEX membrane in preventing peridural fibrosis after spinal surgeries in rats [55]. Seprafilm or Gore-Tex was applied to a laminectomy defect overlying the dura mater and evaluated 2 months later. Both membranes facilitated access to the epidural space and provided a reduction in the amount of tissue adhering to the dura mater. There was a statistically significant decrease in the peridural adhesions with the use of Seprafilm when compared with the Gore-Tex membrane. The second study evaluated rats that underwent hemilaminectomy at L4 and L5 followed by treatment with Seprafilm or Interceed [56]. Both agents significantly reduced epidural fibrosis and dural adhesions at 4 and 8 weeks evaluation. The third study compared ADCON-L to Seprafilm in a rat laminectomy model. Both products were found to be effective in reducing peridural adhesions [54].

Seprafilm has also been evaluated to determine its effects on peripheral nerves, in models, both with and without nerve injury [70]. In the former, Seprafilm was placed either superficially to the nerve or wrapped circumferentially around the nerve. In both cases, Seprafilm had no effect on the nerve at evaluation times up to 6 weeks, as assessed by nerve compression, nerve fibrosis or inflammation, foreign body reaction, or nerve ultrastructure, vascularity, and histomorphology. It was also noted that there were qualitatively fewer adhesions at the operative site, with preservation of the surgical plane medial to the gluteal muscle. In the nerve injury model in which the sciatic nerve was severed and then repaired, Seprafilm treatment was associated with less perinural adhesions. The histomorphological findings favored Seprafilm at the initial evaluation period (18 days) and control at the final evaluation period (42 days), although no functional outcome differences were identified [70].

#### Opthalmic surgery

In a preclinical study, Seprafilm has been used to prevent conjunctival adhesions after strabismus surgery [57]. Seprafilm was applied to the eyes of New Zealand rabbits after dissection in the area of the superior fornix and inferior conjunctival limbus. Six weeks after the initial procedure, eyes in which Seprafilm was applied showed significantly less fibrosis, both at the area of superior rectus resection (*P* < 0.046) and the area of conjunctival dissection (*P* < 0.015) as compared to the control areas. No increase in inflammation was observed at the areas of application of Seprafilm. In other ophthalmic studies, adhesion development in rabbits was reduced by Seprafilm after creation of either conjunctival flaps or when applied above and below scleral flaps [71]. Additionally, Seprafilm application in subconjuctival pockets was associated with a reduction in the mean adhesive force between the sclera and conjunctiva, while placement of Seprafilm on the scleral flap after trabeculectomy resulted in a larger subconjuctival space and reduced intraocular pressure as compared to control rabbits [72]. Seprafilm was subsequently used experimentally for patching of small retinal breaks in a rabbit model of rhegmatogenous retinal detachment [73]. In treated animals, retinal reattachments were identified, as contrasted to proliferative vitreoretinopathy in the control group.

#### Otologic surgery

Adhesions in the middle ear can contribute to conductive hearing loss, as well as tympanic membrane retraction. In a study of guinea pigs, abrasion of the middle ear mucosa through a myringotomy incision was used to create adhesions and evaluate their reduction by Seprafilm [74]. In contrast to control animals, those that had received Seprafilm packing at the conclusion of the surgical procedure had no middle ear cleft adhesions and no differences in auditory brainstem responses at 3 or 6 weeks after surgery [74].

## Human investigational uses of Seprafilm

### Intrauterine

In a prospective, blinded, randomized, controlled study, Seprafilm was studied for adhesion prevention after the suction and/or curettage evacuation of the uterus in 150 women (Table 1) [75]. Seprafilm was applied following the procedure. One Seprafilm membrane was cut into two equal pieces, then rolled and inserted through the end cervical canal into the endometrial cavity. The end point of the study was pregnancy or demonstration of adhesions on hysterosalpingography. Among the women with no prior history of dilatation and curettage (D&C), 100 % of Seprafilm-treated women achieved pregnancy within 8 months of the procedure, compared with 54 % of the control women who became pregnant during the same interval of time. Of the women with prior history of D&C, 33 % of the Seprafilm-treated women and 22.7 % of the control women became pregnant. Hysterosalpingography in women, who had not become pregnant, revealed adhesions in 10 % of the Seprafilm-treated women and 50 % of the control group of women.

**Table 1:** Investigational clinical publications and calculated effect size

Reference	*N*	Therapeutic area	Favorable Seprafilm outcome	Effect size^a^	Reported *p* value
van der Linden. [76]	19	Cardiac	Median retrosternal adhesions	Cannot be calculated, only median and range reported	NS
Koyuncu et al. [78]	23	Vascular	Internal jugular vein function	n/a	NS
Tsapanos et al. [75]	150	Gynecologic	Incidence of adhesions	2.9	Not reported
		Intrauterine surgery	Pregnancies	4.8	
Sanders et al. [77]	249	Neurologic	15 year old historical control group		
			Successful primary scalenectomy	0.9	0.938
			Successful primary scalenectomy/1st rib resection	0.7	0.634
			Successful reoperation: neurolysis	1.3	0.760
			Successful reoperation: scalenectomy and neurolysis	1.6	0.811
Icinose et al. [84]	n/a	Neurologic	No control group or efficacy	n/a	n/a
Assaf et al. [80]	4	Opthalmic	No control group or efficacy	n/a	n/a
Taban et al. [81]	4	Opthalmic	No control group or efficacy	n/a	n/a
Shibata et al. [82]	1	Opthalmic	No control group	n/a	n/a
Filler et al. [79]	239	Orthopedic	No safety, efficacy or control	n/a	n/a
Caylan et al. [83]	21	Otologic	No control group	n/a	n/a

^a^For continuous outcomes, effect size is the (control group mean–the Seprafilm mean) divided by the pooled standard deviation for the two groups [ES = (M1−M2)/pooled SD], and for binomial outcomes, effect size is the odds ratio or the ratio of the odds of a success for the Seprafilm group to the odds of a success for the control group [ES = (ad)/(bc)]

### Cardiac surgery

In a prospective clinical study in patients undergoing coronary artery bypass graft surgery, the median percentage of retrosternal adhesions in the group that received Seprafilm was 53 % (range 27–88 %) versus 71 % (range 18–90 %) in patients who did not receive Seprafilm. This difference was not statistically significant [76].

### Thoracic surgery

Seprafilm was utilized in 249 subjects with neurogenic thoracic outlet syndrome [77]. The barrier was placed over the fine nerve roots of the bronchial plexus at the conclusion of the supraclavicular thoracic outlet decompression procedure, in an attempt to reduce postoperative scarring. Success rates for primary operations for scalenectomy with or without resection of the first rib was 70 and 74 %, respectively, and 78 % for reoperative procedures. These results were no different than historical controls, although reoperations demonstrated a trend towards improvement in symptoms. In ten patients who underwent reoperations, it was thought there were reduced adhesions of the fat pad to the nerve root, thereby facilitating identification of the nerve roots.

### Vascular surgery

In a small study of individuals undergoing functional neck dissection, the impact of wrapping the jugular vein with Seprafilm was evaluated [78]. Three months postoperatively, Doppler ultrasound evaluation of internal jugular vein function identified no differences in the healing process.

### Orthopedic surgery

Seprafilm was used as an adhesiolytic agent in patients who underwent surgical treatment for release of sciatic nerve entrapment in the pelvis or at the level of the ischial tuberosity [79]. No safety or efficacy assessments were made.

### Opthalmic surgery

Seprafilm has been successfully used in four patients undergoing strabismus surgery. Seprafilm was inserted over the muscle without complication. The authors reported the application of Seprafilm to be safe in patients undergoing repeated strabismus surgery [80]. In another report, Seprafilm was applied in four subjects (six sites total) with orbital fractures and entrapped orbital soft tissue [81]. Seprafilm was placed over the fracture in these “trap doors”, with no subsequent need for reoperation and with no complications. Lastly, Seprafilm was used in one patient with neovascular glaucoma who underwent bilateral trabeculectomy. The authors reported that in this patient, trabeculotomy adjunct use of Seprafilm was effective in controlling intraocular pressure, maintenance of scleral flap, and prevention of adhesions between the scleral flap and conjunctiva [82].

### Otologic surgery

In a case series of 21 patients with middle ear and or mastoid cholesteatoma, undergoing staged tympanoplasty with mastoidectomy, Seprafilm was applied in the mastoid cavity [83]. Seprafilm was reported to result in the preservation of the aeration of the mastoid cavity while preventing retraction of postauricular skin. No adverse reactions were reported.

### Neurosurgery

Use of Seprafilm has been described in a series of 13 patients in whom the barrier was placed between the temporal muscle and the dura at the time of cranioplasty for external decompression for brain swelling [84]. Such use was identified by the authors as facilitating subsequent cranioplasties, by decreasing bleeding and operative time, as well as injuries to the temporal muscle or dura.

## Summary

Seprafilm has been extensively studied in intraperitoneal clinical trials, demonstrating safety and efficacy in reducing postoperative adhesion development after intra-abdominal surgery. This report summarizes preclinical studies demonstrating the time course and mechanism of Seprafilm degradation. Additionally, key animal studies involving procedures both intra-abdominally and elsewhere throughout the body have been summarized, identifying efficacy and safety at these diverse locations. Results of published human investigational uses at non-abdominal locations throughout the body have also been detailed. These findings provide the opportunity for future randomized, controlled clinical trials of Seprafilm outside the intra-abdominal cavity, and also the foundation to further improve the efficacy of anti-adhesion adjuvants, perhaps by using barriers (which function separating traumatized surfaces during mesothelial repair) to also serve as a device for local delivery of drugs and/or biologics.
